# Risk factors for spontaneous miscarriage above 12 weeks or premature delivery in patients undergoing cervical polypectomy during pregnancy

**DOI:** 10.1186/s12884-019-2710-z

**Published:** 2020-01-09

**Authors:** Kaori Fukuta, Satoshi Yoneda, Noriko Yoneda, Arihiro Shiozaki, Akitoshi Nakashima, Takashi Minamisaka, Johji Imura, Shigeru Saito

**Affiliations:** 1 Department of Obstetrics and Gynecology, University of Toyama, 2630 Sugitani, Toyama, 930-0194 Japan; 2 Department of Diagnostic Pathology, Graduate School of Medicine and Pharmaceutical Sciences, University of Toyama, 2630 Sugitani, Toyama, 930-0194 Japan

**Keywords:** Cervical polyp, Genital bleeding, Miscarriage, Polypectomy, Preterm birth

## Abstract

**Background:**

It currently remains unknown whether the resection of cervical polyps during pregnancy leads to miscarriage and/or preterm birth. This study evaluated the risk of spontaneous PTB below 34 or 37 weeks and miscarriage above 12 weeks in patients undergoing cervical polypectomy during pregnancy.

**Methods:**

This was a retrospective monocentric cohort study of patients undergoing cervical polypectomy for clinical indication. Seventy-three pregnant women who underwent polypectomy were selected, and risk factors associated with miscarriage above 12 weeks or premature delivery below 34 or 37 weeks were investigated. A multivariable regression looking for predictors of spontaneous miscarriage > 12 weeks and PTB < 34 or 37 weeks were performed.

**Results:**

Sixteen patients (21.9%, 16/73) had spontaneous delivery at < 34 weeks or miscarriage above 12 weeks. A univariate analysis showed that bleeding before polypectomy [odds ratio (OR) 7.7, 95% confidence interval (CI) 1.6–37.3, *p* = 0.004], polyp width ≥ 12 mm (OR 4.0, 95% CI 1.2–13.1, *p* = 0.005), the proportion of decidual polyps (OR 8.1, 95% CI 1.00–65.9, *p* = 0.024), and polypectomy at ≤10 weeks (OR 5.2, 95% CI 1.3–20.3, *p* = 0.01) were significantly higher in delivery at < 34 weeks than at ≥34 weeks. A logistic regression analysis identified polyp width ≥ 12 mm (OR 11.8, 95% CI 2.8–77.5, *p* = 0.001), genital bleeding before polypectomy (OR 6.5, 95% CI 1.2–55.7, *p* = 0.025), and polypectomy at ≤10 weeks (OR 5.9, 95% CI 1.2–45.0, *p* = 0.028) as independent risk factors for predicting delivery at < 34 weeks. Polyp width ≥ 12 mm and bleeding before polypectomy are risk factors for PTB < 37 wks.

**Conclusions:**

Our cohort of patients undergoing polypectomy in pregnancy have high risks of miscarriage or spontaneous premature delivery. It is unclear whether these risks are given by the underlying disease, by surgical treatment or both. This study establishes clinically relevant predictors of PTB are polyp size> 12 mm, bleeding and first trimester polypectomy. PTB risks should be exposed to patients and extensively discussed with balancing against the benefits of intervention in pregnancy.

## Background

Cervical polyps cause genital bleeding and vaginal discharge. There may also be a risk of cervicitis and pelvic inflammatory disease due to chronic inflammation [1–4]. In non-pregnant women, it is generally accepted that cervical polyps need to be removed because of discomfort or recurrent bleeding. Furthermore, lesions need to be examined histologically to confirm whether they are malignant [5–10].

On the other hand, among pregnant women, the resection of cervical polyps is generally recommended for patients with clinical symptoms, such as genital bleeding or vaginal discharge [11]. A previous case report showed that the gestational period was prolonged by the removal of cervical polyps associated with massive genital bleeding in the first trimester [12]. However, there are no guidelines on whether cervical polyps found during pregnancy need to be removed. Moreover, it currently remains unclear whether cervical polyps during pregnancy are a risk factor for miscarriage or spontaneous preterm birth (SPTB).

Cervical polyps have various histologies. Endocervical polyps, the most common lesion, are hyperplastic protrusions of the endocervical folds. During pregnancy, endocervical polyps may show focal stromal pseudodecidual changes. Difficulties are sometimes associated with distinguishing between a decidualized endocervical polyp and a fragment of the decidua extruding into the endocervical area; therefore, they are often collectively termed “decidual polyps” [4, 13]. Tokunaka et al. reported that decidual polyps were associated with a higher risk of spontaneous miscarriage and SPTB than endocervical polyps in pregnant women, and, thus, it may be safer to avoid removing cervical polyps during pregnancy, except those suspected to be malignant [14].

As a clinical feature, incidence of SPTB due to intrauterine inflammation and/or infection is greater in earlier preterm pregnancy [15–17].. We speculated that the presence of cervical polyps is a risk factor for ascending inflammation and/or infection into the uterine cavity, and we have performed polypectomy in an attempt to reduce the risk of spontaneous miscarriage and preterm birth (PTB). The aim of the present study was to retrospectively investigate the risk of miscarriage ≥12 weeks and SPTB, particularly delivery at < 34 weeks of gestation without late PTB, in patients undergoing cervical polypectomy in pregnancy.

## Methods

### Study population

Seventy-three patients who underwent cervical polypectomy during pregnancy at Toyama University Hospital between January 2003 and June 2017 were selected by the ICD code of the cervical polyp (N841). Indication was given by individual physicians based on their assessment and define risk factors for spontaneous miscarriage or preterm delivery below 34 or 37 weeks. Inclusion criteria were outpatients, singleton pregnancies, asymptomatic patients in whom cervical polyps were incidentally detected, or those with genital bleeding from cervical polyps. Pregnant women who delivered because of an obstetric indication, such as preeclampsia, placental previa, gestational diabetes mellitus, and abruption of the placenta, were not included. All patients were pathologically confirmed to have cervical polyps. At our hospital, we recommend the resection of cervical polyps because they may cause inflammation and/or infection, resulting in PTB [18, 19]. We explained the procedure to patients and performed polypectomy after obtaining written informed consent. The present study was approved by the Ethics Committee of Toyama University Hospital (No.25–74).

### Management for cervical polyps during pregnancy

Cervical polyps > 5 mm were resected with or without clinical symptoms, such as genital bleeding. In patients with small cervical polyps (< 5 mm), polypectomy was considered at the discretion of the attending obstetricians.

Although the procedure for polypectomy differs [5, 11, 20], we resected cervical polyps by twisting using Kelly and Pean forceps. In the case of larger cervical polyps, ligation at the root of the polyp was performed following by its resection; however, it was not possible to observe the root of the cervical polyp using hysteroscopy [5]. No antibiotics were administered after polypectomy.

Resected cervical polyps were diagnosed by a pathologist and grouped into three types: endocervical polyps, decidual polyps, and others. Endocervical polyps were defined as polyps lined by a benign endocervical epithelium covering a fibrovascular core, and decidual polyps were defined as polyps with decidual or pseudodecidual stroma, including fragments of decidua and decidualized endocervical polyps [4, 13].

Histological chorioamnionitis and funisitis were evaluated according to Blanc’s classification [21].

### Study procedures

This was a retrospective cohort study. Demographic and clinical data (maternal age, nulliparity, smoking, previous second trimester loss, previous history of full-term delivery, other maternal diseases, uterine anomalies, use of vaginal progesterone, infertility treatment, previous history of PTB at < 34 weeks, bleeding before polypectomy, Nugent score of vaginal secretion, gestational age at polypectomy, polypectomy by twisting, polyp width, decidual polyps, number of leukocytes in polyps, histological chorioamnionitis ≥ stage 2, and funisitis [21]) were collected and analysed as covariates within the statistical model for the prediction of miscarriage and/or PTB.

‘Bleeding before polypectomy’ was defined as bleeding from a polyp that was directly confirmed under direct vision, or bleeding from adhesive root or stem of the cervical polyp was strongly speculated, because there is no founding, such as placental abruption, previa, or subchorionic haemorrhage by ultrasonography. The Nugent score is one of the diagnostic methods used for bacterial vaginosis (BV) and involves examining Gram-stained smears of vaginal discharge [22]. A previous study reported that pregnant women with BV are at a higher risk of PTB [23].

The number of leukocytes in cervical polyps was previously associated with cervical inflammation/infection [19, 24]. Leukocytes were counted using haematoxylin-eosin-stained specimens. We counted leukocytes in three high power fields (HPFs), and the average was calculated in each case [25].

### Statistical analysis

To identify the clinical variables associated with a significant difference between delivery at < 34 weeks and delivery at ≥34 weeks of gestation, univariate analyses were performed using the *χ*^2^-test, Student’s *t*-test, or Mann–Whitney *U* test where appropriate. A multiple logistic regression analysis was performed to identify independent risk factors that correlate with delivery at < 34 weeks of gestation (including miscarriage at ≥12 weeks). Diagnostic values for predicting delivery at < 34 weeks were calculated using significant risk factors. We also investigated independent risk factors for delivery < 37 weeks of gestation; however, only 4 patients delivered at 34 to 36 weeks. Receiver operating characteristic (ROC) curves were drawn to correlate with delivery at < 34 weeks, and the relationship between clinical risk factors and delivery at < 34 weeks was assessed using a multiple logistic regression analysis. The odds ratio (OR) and 95% confidence intervals (CI) were also calculated. All analyses were performed using statistical analysis software (JMP, version 11.2.0; SAS Institute Inc., Tokyo, Japan). A *p* value < 0.05 was regarded as significant.

## Results

**Fig. 1:**
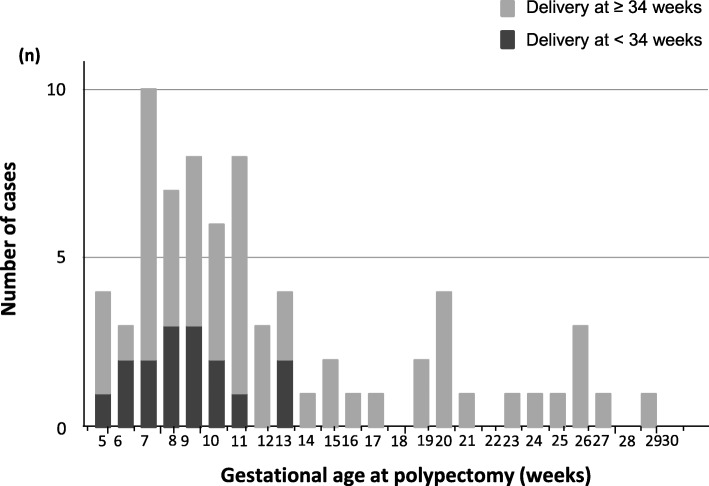
Number of patients according to gestational age at polypectomy (weeks). Fifty-three patients (72.6%, 53/73) underwent polypectomy at < 14 weeks of gestation. Among patients who delivered at < 34 weeks, polypectomy was performed before 13 weeks of gestation

**Fig. 2:**
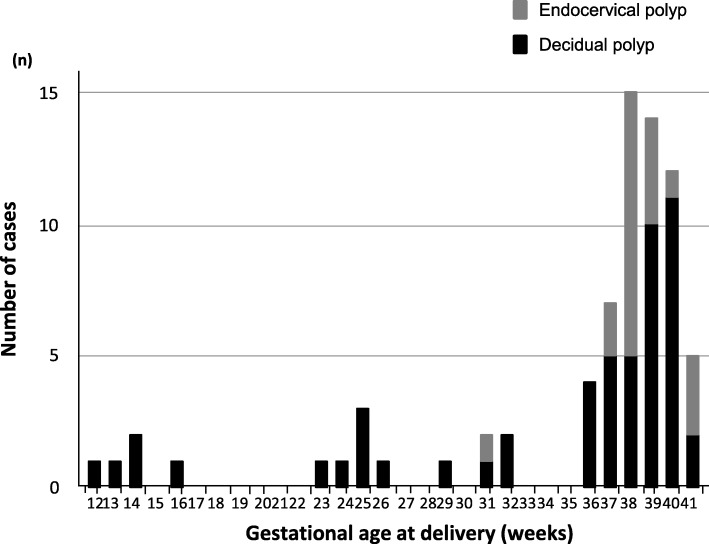
Number of patients according to gestational age at delivery (weeks). Sixteen patients (21.9%, 16/73) delivered at < 34 weeks of gestation, and the frequency of decidual polyps was 93.7% (15/16)

Among 73 patients, 53 (72.6%, 53/73) had undergone cervical polypectomy at < 14 weeks of gestation (Fig. 1). Sixteen patients (21.9%, 16/73) delivered at < 34 weeks and 20 (27%, 20/73) at < 37 weeks, including five miscarriages (6.8%, 5/73) (Fig. 2).

**Fig. 3:**
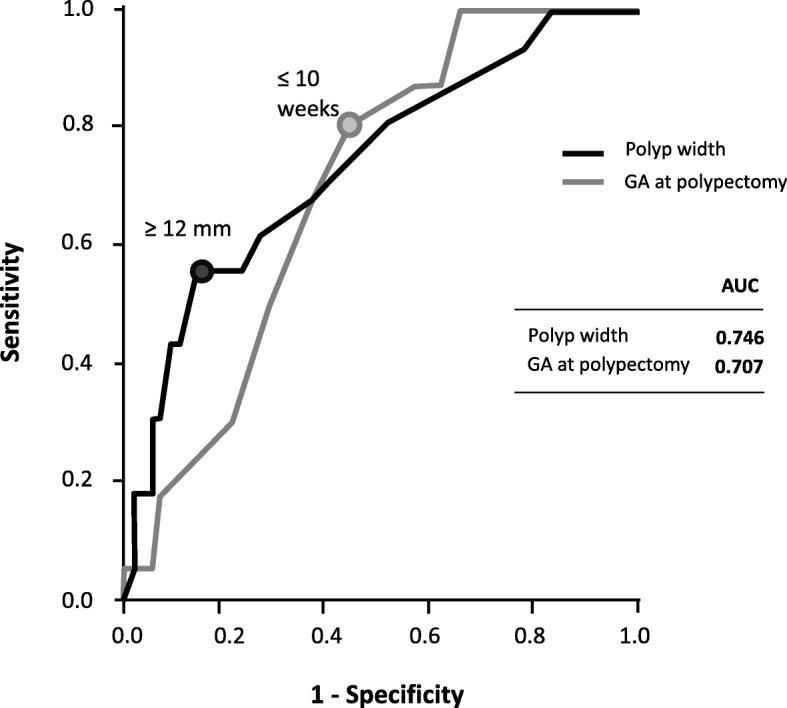
Receiver operating characteristic (ROC) curves of each risk factor for delivery at < 34 weeks or miscarriage > 12 weeks of gestation. Cut-off values were polyp width ≥ 12 mm (AUC = 0.746) and gestational age ≤ 10 weeks at polypectomy (AUC = 0.707)

Table 1 shows a comparison of clinical features between PTB, PTB + Miscarriage ≥12 weeks, and Term delivery. Polyp width (11.1 ± 8.8 mm and 12.5 ± 8.5 mm) and the proportion of decidual polyps (93.3 and 95.0%) in the PTB and PTB + Miscarriage groups were significantly different from those in the Term delivery group (6.8 ± 3.9 mm and 62.2%, respectively). Table 2 shows a comparison of clinical features between delivery at < 34 weeks (Delivery at < 34 weeks group) and delivery at ≥34 weeks (Delivery at ≥34 weeks group). The proportion of genital bleeding before polypectomy (87.5%), polyp width (11.1 ± 8.8 mm), the proportion of decidual polyps (93.7%), and histological chorioamnionitis (61.5%) in the Delivery at < 34 weeks group were significantly higher than those (47.4%, 7.4 ± 4.4 mm, 64.9, and 20.0%, respectively) in the Delivery at ≥34 weeks group. On the other hand, gestational age at polypectomy (8.6 ± 2.3 weeks) in the Delivery at < 34 weeks group was significantly lower than that (11.7 ± 6.6 weeks) in the Delivery at ≥34 weeks group. Polyp width ≥ 12 mm (AUC = 0.746) and ≤ 10 weeks of gestation at polypectomy (AUC = 0.707) were cut-off values that correlated with delivery at < 34 weeks of gestation using the ROC curve (Fig. 3).

**Table 1:** Comparison of clinical features: between PTB and Term delivery, between PTB + Miscarriage and Term delivery

Clinical feature	PTB	PTB + Miscarriage	Term delivery
	(*N* = 15)	(*N* = 20)	(*N* = 53)
Age	33.2 ± 5.3	32.5 ± 5.2	34.3 ± 3.4
Nulliparity (%)	40.0	45.0	50.9
Smoking (%)	6.6	5.0	1.8
Previous second trimester loss (%)	26.6	35.0^**^	13.2
History of full-term delivery (%)	50.0	40.0	57.1
Other maternal diseases (%)	6.6	10.0	30.1
Uterine anomalies (%)	6.6	15.0	0.8
Use of vaginal progesterone (%)	0	0	0
Infertility treatment (%)	13.3	10.0	18.8
History of PTB (at < 34 weeks) (%)	40.0	33.3	13.8
Bleeding before polypectomy (%)	73.3	75.0^**^	49.0
Nugent score (points)	0 (0–8)	0 (0–8)	0 (0–10)
GA at polypectomy (weeks)	9 (5–25)	9 (5–25)	11 (5–29)
Polypectomy by twisting (%)	80.0	70.0^**^	90.5
Polyp width (mm)	11.1 ± 8.8^*^	12.5 ± 8.5^**^	6.8 ± 3.9
Decidual polyps (%)	93.3^*^	95.0^**^	62.2
Leukocytes in polyps (/HPF)	275 ± 128	280 ± 147	314 ± 201
Prelabour rupture of membranes (%)	26.6	25.0	11.3
h-CAM ≥ stage 2 (%)	72.7^*^	50.0	20.0
Funisitis (%)	45.4	31.2	24.0
Offspring GA at birth (weeks)	29.8 ± 4.8^*^	25.8 ± 8.3^**^	38.8 ± 1.2
Offspring birth weight (g)	1540 ± 774^*^	1365 ± 875^**^	3027 ± 392

Data indicate the mean ± standard deviation or median (range), *PTB* preterm birth, *GA* gestational age, *HPF* high power field, *h-CAM* histological chorioamnionitis, *N/A* not available, ^*^: Significant difference between PTB and Term delivery, *p* <  0.05, ^**^: Significant difference between PTB + Miscarriage and Term delivery, *p* <  0.05

**Table 2:** Comparison of clinical features between delivery at < 34 weeks or miscarriage > 12 weeks and delivery at ≥34 weeks of gestation

			(*N* = 73)
Clinical feature	Delivery at < 34 weeks or miscarriage > 12 weeks	Delivery at ≥ 34 weeks	*p-*value
	(*N* = 16)	(*N* = 57)	
Age	32.6 ± 4.9	34.1 ± 3.7	0.283
Nulliparity (%)	50.0	49.1	0.950
Smoking (%)	6.2	1.7	0.503
Previous second trimester loss (%)	37.5	14.0	0.094
History of full-term delivery (%)	33.3	56.4	0.302
Other maternal diseases (%)	12.5	28.0	0.201
Uterine anomalies (%)	18.7	5.2	0.215
Use of vaginal progesterone (%)	0	0	N/A
Infertility treatment (%)	6.2	17.5	0.264
History of PTB (at < 34 weeks) (%)	25.0	10.5	0.137
Bleeding before polypectomy (%)	87.5^*^	47.4	0.004
Nugent score (points)	0 (0–8)	0 (0–5)	0.640
GA at polypectomy (weeks)	9^*^ (5–13)	11 (5–29)	< 0.001
Polypectomy by twisting (%)	68.7	87.7	0.070
Polyp Width (mm)	12.8 ± 7.7^*^	7.4 ± 4.4	0.012
Decidual polyps (%)	93.7^*^	64.9	0.024
Leukocytes in polyps (/HPF)	311 ± 147	286 ± 198	0.683
h-CAM ≥ stage 2 (%)	61.5^*^	20.0	0.010
Funisitis (%)	38.5	24.0	0.351

Data indicate the mean ± standard deviation or median (range), *PTB* preterm birth, *GA* gestational age, *HPF* high power field, *h-CAM* histological chorioamnionitis, *N/A* not available, ^*^: *p* < 0.05.

Table 3 shows the risk factors identified by a univariate analysis and a multiple logistic regression analysis for delivery at < 34 weeks (a) or < 37 weeks (b) of gestation in patients who had undergone polypectomy. By a univariate analysis, polyp width (≥ 12 mm), bleeding before polypectomy, polypectomy at ≤10 weeks, and the proportion of decidual polyps were significantly different in the delivery at < 34 weeks and < 37 weeks group. The independent risk factors identified by a multiple logistic regression analysis for delivery at < 34 weeks and delivery at < 37 weeks were polyp width ≥ 12 mm [11.8 (2.5–77.5), *p* = 0.001], genital bleeding before polypectomy [6.5 (1.2–55.7), *p* = 0.025], and polypectomy at ≤10 weeks of gestation [5.9 (1.2–45.0), *p* = 0.028] in the study of delivery at < 34 weeks. Polyp width ≥ 12 mm [6.5 (1.6–30.9), *p* = 0.008] and polypectomy at ≤10 weeks of gestation [4.0 (1.1–17.6), *p* = 0.032] were independent risk factors for delivery at < 37 weeks.

**Table 3:** Risk factors identified by a univariate analysis and a multiple logistic regression analysis for delivery < 34 weeks^§^or < 37 weeks^∮^of gestation in patients who had undergone polypectomy

	Odds ratio	
	crude (95% CI)	adjusted (95% CI)
(a) Delivery < 34 weeks^§^of gestation		
Polyp width (≥ 12 mm)	4.0* (1.2–13.1)	11.8* (2.5–77.5)
Bleeding before PP	7.7* (1.6–37.3)	6.5* (1.2–55.7)
PP (at ≤10 weeks)	5.2** (1.3–20.3)	5.9** (1.2–45.0)
Decidual polyps	8.1** (1.00–65.9)	2.8 (0.37–58.1)
History of PTB	2.8 (0.69–11.6)	0.84 (0.11–5.3)
(b) Delivery < 37 weeks^∮^of gestation		
Polyp width (≥ 12 mm)	6.5* (2.0–21.4)	6.5* (1.6–30.9)
Bleeding before PP	3.1** (1.00–9.80)	1.8 (0.51–7.1)
PP (at ≤10 weeks)	3.9** (1.2–12.3)	4.0** (1.1–17.6)
Decidual polyps	11.5* (1.4–92.7)	1.2 (0.28–5.3)
History of PTB	2.6 (0.69–9.78)	1.4 (0.24–7.2)

^§^Delivery < 34 weeks: PTB < 34 weeks or miscarriage > 12 weeks, crude: The odds ratio calculated by a univariate analysis, adjusted: The odds ratio calculated by a multiple logistic regression analysis, CI: confidence interval, PP: polypectomy, PTB: preterm birth, *: *p* < 0.01, **: *p* < 0.05

^∮^Delivery < 37 weeks: PTB < 37 weeks or miscarriage > 12 weeks, crude: The odds ratio calculated by a univariate analysis, adjusted: The odds ratio calculated by a multiple logistic regression analysis, CI: confidence interval, PP: polypectomy, PTB: preterm birth, *: *p* < 0.01, **: *p* < 0.05

Diagnostic values for predicting delivery at < 34 weeks were sensitivity of 56.2% and specificity of 86.0% for polyp width (≥ 12 mm), 87.5 and 52.6% for genital bleeding before polypectomy, and 81.2 and 56.1% by polypectomy at ≤10 weeks, respectively. The positive predictive value for the combination of these three factors was 71.4% (Table 4).

**Table 4:** Diagnostic value for predicting delivery at < 34 weeks [< 37 weeks] of gestation (*n* = 73)

	Sensitivity (%)	Specificity (%)	PPV (%)	NPV (%)
Polyp width (≥ 12 mm)	56.2 [50.0]	86.0 [86.7]	52.9 [58.8]	87.5 [82.1]
Bleeding before polypectomy	87.5 [75.0]	52.6 [50.9]	34.1 [36.5]	93.7 [84.3]
Polypectomy at ≤10 weeks	81.2 [75.0]	56.1 [56.6]	34.2 [39.4]	91.4 [85.7]
Width & bleeding	43.7 [35.0]	93.0 [90.5]	63.6 [58.3]	85.5 [78.6]
Width & polypectomy	37.5 [35.0]	94.7 [96.2]	66.7 [77.7]	84.4 [79.6]
Bleeding & polypectomy	75.0 [60.0]	75.4 [73.4]	46.1 [46.1]	91.5 [82.9]
Width & bleeding & polypectomy	31.2 [25.0]	96.5 [96.2]	71.4 [71.4]	83.3 [77.2]

Values in brackets [] are the results of risk factors for delivery < 37 weeks of gestation

*PPV* positive predictive value, *NPV* negative predictive value

## Discussion

### Clinical significance

Based on the risk of ascending inflammation/infection due to cervical polyps during pregnancy, we empirically performed cervical polypectomy to prevent PTB. However, the frequency of delivery at < 34 weeks of gestation was still 21.9%, which was higher than the general PTB rate at < 37 weeks (5.7%) in Japan. Although an analysis to identify risk factors for delivery at < 34 weeks was limited to cases of cervical polypectomy, “polyps ≥ 12 mm”, “genital bleeding before polypectomy”, and “polypectomy at ≤ 10 weeks” were identified as independent risk factors for delivery at < 34 weeks by a multiple logistic regression analysis. Furthermore, independent risk factors for delivery at < 37 weeks were “polyps ≥12 mm” and “polypectomy at ≤10 weeks”.

Cervical polyps may be a risk factor for delivery at < 34 weeks because they are an inflammatory disease [1–4], or polypectomy itself may induce inflammation/infection in the cervix. It currently remains unclear whether the cause of delivery at < 34 weeks was the cervical polyp itself or polypectomy. In addition, although a previous history of PTB is an important risk factor for PTB [26–28], “polyps ≥ 12 mm”, “genital bleeding before polypectomy”, and “polypectomy at ≤ 10 weeks” were identified as stronger risk factors in the present study. The presence of cervical polyps and/or polypectomy during pregnancy may have markedly increased the risk of spontaneous miscarriage or PTB.

It may be preferable not to perform polypectomy on pregnant patients with cervical polyp, particularly with decidual polyps, except for suspected malignancy [14]. It is unclear whether this treatment is beneficial, therefore extreme caution should be applied when indicating such procedure. However, the risk of spontaneous miscarriage or PTB may increase without polypectomy [19].

### Postulated mechanism of PTB

Although multiple pathological processes are associated with SPTB [26, 29, 30], intra-amniotic inflammation and/or infection play a central role [15–17, 31]. In the earlier weeks of gestation before SPTB, severe intra-amniotic inflammation and/or intra-amniotic infection were found to be more frequent [17, 31]. One of the major causes of intra-amniotic inflammation and/or infection is considered to be BV or cervicitis during pregnancy [18].

Cervical polyps themselves comprise an inflammatory disease [1–4] and cause genital bleeding [32]. In pregnant women, genital bleeding is a known risk factor for miscarriage or SPTB [33, 34]. In addition, cervical polyps may affect the cervix, resulting in cervicitis, which is considered to be a risk factor for increasingly severe inflammation and/or infection in the uterine cavity [35–37], resulting in intra-amniotic inflammation and/or infection and induced miscarriage or SPTB. Levin et al. postulated that the mechanisms underlying decidual trauma and placental abruption are part of the aetiology of PTB when cervical polypectomy is performed during pregnancy [10].

A clearer understanding of the mechanisms underlying PTB will contribute to decisions regarding whether cervical polyps need to be resected during pregnancy.

### Limitation and strengths

The following are potential limitations of the present study. (i) The clinical data presented were gathered from a single hospital using a small sample (73 cases); therefore, further studies with a larger patient population may increase the significance of the results obtained. (ii) Since cervicitis was not evaluated using inflammatory markers, such as cervical mucus IL-8, before and after polypectomy, it currently remains unknown whether inflammation in the cervix was induced by the cervical polyp itself or by its resection. (iii) Inflammation and/or infection associated with a cervical polyp may affect the uterus, thereby resulting in delivery at < 34 weeks; however, it was not possible to prove that the cervical polyp itself or polypectomy directly resulted in increasingly severe inflammation and/or infection in the uterine cavity. (iv) The statistical power of this study is low. (v) Due to the lack of a control group in the present study, it has yet to be clarified whether cervical polyps during pregnancy need to be monitored or resected. (vi) Although vaginal progesterone decreases the risk of preterm birth with a short cervix worldwide [38], vaginal progesterone therapy is not covered by medical insurance in Japan at the moment. So, there was no use of vaginal progesterone in this study.

The following are the strengths of the present study. (i) Although the study population was only polypectomy cases, this is the first study to investigate the relationship between delivery at < 34 weeks and polypectomy during pregnancy, including clinical risk factors. (ii) The present results may be useful when a large cervical polyp, with or without genital bleeding, is found in the first trimester during pregnancy to provide clinical counselling to the patients.

### Proposals for future research

A randomized controlled trial is needed to compare between observations and treatment by the resection of cervical polyps during pregnancy with a sufficient number of cases according to each category such as spontaneous miscarriage or SPTB. In addition, cervical inflammation/infection needs to be evaluated before and after polypectomy to identify whether the cervical polyp itself or polypectomy induces inflammation/infection. Furthermore, the mechanisms by which decidual polyps form during pregnancy need to be elucidated.

## Conclusions

Although cervical polyps are occasionally found during pregnancy, it currently remains unclear whether they need to be resected. When cervical polyps with genital bleeding and/or a large size (width ≥ 12 mm) during pregnancy are resected at ≤10 weeks of gestation, the risk of delivery at < 34 weeks needs to be considered. It also has yet to be established whether cervical polyps during pregnancy need to be monitored or resected; however, the present results will contribute to the better delineation of risk stratification for patients. Although it may be safer for cervical polyps to be managed in a conservative manner during pregnancy [10, 14], when cervical polyps need to be resected for clinical decision due to any reason a risk stratification may be applied based on our risk assessment.

## Data Availability

The datasets used during the present study are available from the corresponding author on reasonable request.

## Abbreviations

BV: Bacterial vaginosis
CIs: Confidence intervals
HPFs: High power fields
OR: Odds ratio
PTB: Preterm birth
ROC: Receiver operating characteristic
SPTB: Spontaneous preterm birth;
